# The Importance of Bacterial and Viral Infections Associated with Adult Asthma Exacerbations in Clinical Practice

**DOI:** 10.1371/journal.pone.0123584

**Published:** 2015-04-22

**Authors:** Motoyasu Iikura, Masayuki Hojo, Rikiya Koketsu, Sho Watanabe, Ayano Sato, Haruka Chino, Shoki Ro, Haruna Masaki, Junko Hirashima, Satoru Ishii, Go Naka, Jin Takasaki, Shinyu Izumi, Nobuyuki Kobayashi, Sachiko Yamaguchi, Susumu Nakae, Haruhito Sugiyama

**Affiliations:** 1 Department of Respiratory Medicine, National Center for Global Health and Medicine, Tokyo, Japan; 2 Laboratory of Systems Biology, Center for Experimental Medicine and Systems Biology, The Institute of Medical Science, The University of Tokyo, Tokyo, Japan; 3 Precursory Research for Embryonic Science and Technology, Japan Science and Technology Agency, Saitama, Japan; Kliniken der Stadt Köln gGmbH, GERMANY

## Abstract

**Background:**

Viral infection is one of the risk factors for asthma exacerbation. However, which pathogens are related to asthma exacerbation in adults remains unclear.

**Objective:**

The relation between various infections and adult asthma exacerbations was investigated in clinical practice.

**Methods:**

The study subjects included 50 adult inpatients due to asthma exacerbations and 20 stable outpatients for comparison. The pathogens from a nasopharyngeal swab were measured by multiplex PCR analysis.

**Results:**

Asthma exacerbations occurred after a common cold in 48 inpatients. The numbers of patients with viral, bacterial, or both infections were 16, 9, and 9, respectively. The dominant viruses were rhinoviruses, respiratory syncytial virus, influenza virus, and metapneumovirus. The major bacteria were *S*. *pneumoniae* and *H*. *influenzae*. Compared to pathogen-free patients, the patients with pathogens were older and non-atopic and had later onset of disease, lower FeNO levels, lower IgE titers, and a higher incidence of comorbid sinusitis, COPD, or pneumonia. Compared to stable outpatients, asthma exacerbation inpatients had a higher incidence of smoking and comorbid sinusitis, COPD, or pneumonia. Viruses were detected in 50% of stable outpatients, but a higher incidence of rhinovirus, respiratory syncytial virus, and metapneumovirus infections was observed in asthma exacerbation inpatients. *H*. *influenzae* was observed in stable asthmatic patients. Other bacteria, especially *S*. *pneumoniae*, were important in asthma exacerbation inpatients.

**Conclusion:**

Viral or bacterial infections were observed in 70% of inpatients with an asthma exacerbation in clinical practice. Infection with *S*. *pneumoniae* was related to adult asthma exacerbation.

## Introduction

Inhaled antigens including house dust mite are known to be the major cause of asthma exacerbation. Other triggers are cold air, smoking, drinking alcohol, exercise, the use of non-steroidal anti-inflammatory drugs, and viral infection [1–4]. Several viral infections induce asthma exacerbation. Respiratory syncytial virus (RSV) and human rhinovirus (HRV) are known to be major causes of asthma exacerbations in children [5–7]. Infection with these viruses in infants was associated with a higher incidence of asthma onset [8, 9]. The prevalence of virus detection in adult asthmatic exacerbation was reported to the range of 41–78% [10]. Among these viruses, HRV infection is a frequent cause of exacerbations in adults with asthma and a cold [11] HRV infection causes asthma due to its potential for a Th2-biased response [12, 13]. Influenza virus (IF) infection also induces asthma attacks in adults [10, 14]. The prevalence of IF infection with asthmatic exacerbation in adults is higher than that in children [10]. However, whether other viruses induce asthma exacerbations in adults remains unknown.

There are few reports regarding the relationships between asthma attacks and bacterial infections [1–4, 15, 16]. Infection with atypical bacteria, including *Mycoplasma pneumoniae*, *Chlamydophila pneumoniae*, and *Coxiella burnetti*, has been reported to be related to asthma attacks [17–19]. However, it is controversial whether the atypical bacteria are truly related with adult asthmatic exacerbation or not [10].

On *in vitro* examination, many viruses and bacteria are capable of activating several allergic inflammatory cells, including mast cells, eosinophils, bronchial epithelial cells, and smooth muscle cells. Mast cells express toll-like receptor (TLR) 4 on their surface [20]. TLR4 is a receptor for bacterial lipopolysaccharide (LPS), as well as a major receptor for RSV [21]. After stimulation of TLR4 ligands, mast cells induce a subset of genes that include a Th2 cytokine and chemokines that recruit Th2 cells and eosinophils [20]. Eosinophils express TLR7 and TLR8 [22]. TLR7 activation inhibits viral replication in the lung and prevents virus-induced airway hyperreactivity [23]. Bronchial epithelial cells express ICAM-1, which is upregulated by HRV infection [24, 25]. Smooth muscle cells express functional TLR2, TLR3, TLR4, TLR7, and NOD1 with induction of IL-6, IL-8, and GM-CSF release and upregulation of ICAM-1 by HRV infection [26, 27].

Previous studies regarding asthma and infection in uncomplicated asthmatic patients have been limited. Patients with comorbid pneumonia or chronic obstructive pulmonary disease (COPD) are excluded from usual clinical asthma studies. In our previous reports, 11.3% of asthmatics were found to have COPD in clinical practice [28]. The incidence of pneumonia is usually high in asthmatic patients. In the present study, the associations between several infections and adult asthma exacerbations were evaluated in clinical practice. A total of 50 adult inpatients with asthma attacks with or without comorbid pneumonia or COPD were recruited. Nasopharyngeal swabs were obtained from these patients, and several viral and bacterial infections were detected by multiplex PCR analysis. A comprehensive analysis to examine the relation of various infections on adult asthma exacerbation in clinical practice was performed.

## Materials and Methods

### Ethics statement

This study was approved by the Institutional Review Board of the National Center for Global Health and Medicine, and written informed consent was obtained from each participant. This study was conducted according to the principles expressed in the Declaration of Helsinki.

### Study design

Eligible patients were aged over 18 years and had a clinical diagnosis of asthma supported by one or more other characteristics: variability in peak expiratory flow of more than 20%; airway reversibility by inhaled β2 agonist; hyperresponsiveness to methacholine challenge; and recurrent dyspnea episodes with wheezing.

Fifty-five adult asthmatic patients were admitted to our hospital due to asthma exacerbations from May 2011 to December 2012. Asthmatic inpatients who were admitted to our hospital for other disease or who did not require systemic steroid treatment were excluded. Among 55 patients, 50 asthmatics were enrolled in this study with written informed consent. In comparison 20 stable asthmatic patients without any asthmatic attack in previous one year were recruited from the outpatient clinic in our hospital. These patients had no recent common cold in previous 2 weeks.

### PCR analysis

Multiplex PCR analysis to detect 15 major respiratory viruses and 5 bacteria was performed using nasopharyngeal swabs taken from these patients. In brief, the nasopharyngeal swab was taken from each patient as soon as possible after hospital admission. The nasopharyngeal swab was dipped in PBS buffer, and the appropriate volume of RNAlater (Ambion, Austin, TX, USA) solution for extraction of RNA was added. Multiplex PCR was performed using these samples according to the manufacturer’s instructions (RV15OneStepACE Detection kit and PneumoBacter ACE Detection kit, SeeGene, Gaithersburg, MD, USA). The PCR products were electrophoresed through a 2% agarose gel and visualized with ethidium bromide. At the same time, the sputum culture from patients was performed for detection of bacterial infection.

### Statistical analyses

Differences between two groups were assessed using Pearson’s χ^2^ test, Student’s *t*-test or Mann-Whitney’s U test. Additional analysis was conducted by multiple logistic regression model. Data analyses were performed with SPSS statistics version 17.0.0 (IBM Japan, Tokyo, Japan).

## Results

### Patients’ characteristics

Fifty patients were admitted to our hospital for asthma exacerbations. These patients’ characteristics are shown in Table 1. Their mean age was 57.9 years, and the average duration of asthma was 20.6 years. Eighty percent of the patients were atopic, 34% were non-smokers, and current smokers accounted for 36%. The comorbidities of the patients included sinusitis (32%), COPD (16%), and pneumonia (6%). The patients had poor asthma control (ACQ 3.7 ± 1.2) with high exhaled NO levels (FeNO 51.7 ± 47.6 ppb).

**Table 1:** Patients’ characteristics.

**Asthma exacerbation patients’ characteristics**		**(*n* = 50)**
**Male, n (%)**		19 (38.0)
**Mean age, y (range)**		58 (28–92)
**Mean age at the asthma onset, y (range)**		38 (2–84)
**Atopic asthma, n (%)**		40 (80.0)
**Smoking status**	Non-smoker, n (%)	17 (34.0)
	Ex-smoker, n (%)	15 (30.0)
	Current smoker, n (%)	18 (36.0)
**Co-morbidity**	Sinusitis, n (%)	16 (32.0)
	COPD, n (%)	8 (16.0)
	Pneumonia, n (%)	3 (6.0)
**IgE (IU/mL)**		597 (0–11837)
**ACQ**		3.7 (0.4–5.4)
**FeNO (ppb)**		51.7 (8–225)
	**Asthma exacerbation inpatients**	**Stable outpatients**
**n**	48	20
**Male (%)**	39.6	45.0
**Age (y)**	57.6	60.2
**Asthma onset age (y)**	38.4	39.9
**Duration of disease (y)**	20.2	21.3
**Current smoker(%)**	37.5*	5.0
**Sinusitis (%)**	29.2	15.0
**COPD (%)**	16.7	5.0
**Atopic type (%)**	81.9	84.2
**IgE(IU/mL)**	617.2	574.9
**ACQ**	3.7* †	0.4
**FeNO (ppb)**	51.2	39.4
**Pathogen detection(%)**	70.8	75.0
**Viral detection (%)**	52.1	50.0
**Bacterial detection (%)**	37.5	30.0

*p < 0.05, by Pearson’s χ^2^ test, Student’s *t*-test or Mann-Whitney’s U test.

†p < 0.05, by multiple logistic regression analysis.

Compared to stable outpatients, asthma exacerbation inpatients had higher incidences of smoking and comorbid sinusitis, COPD, and pneumonia. There was no difference in the pathogen detection rate between inpatients and outpatients (Table 1).

### Virus and bacteria detection in asthma exacerbation patients

**Fig 1:**
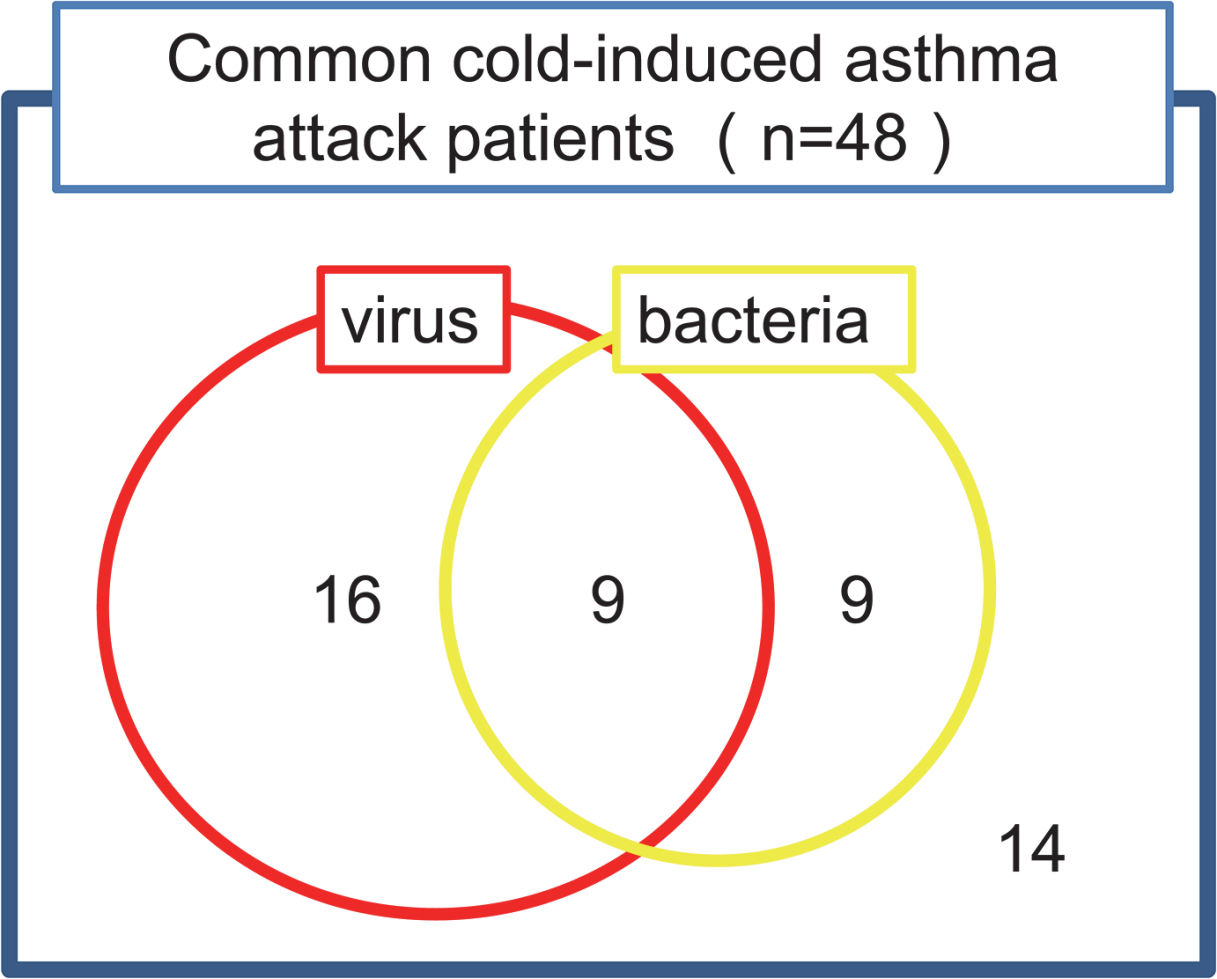
Pathogen detection from asthma exacerbation patients after a common cold.

Of the 50 asthma exacerbation inpatients, 48 suffered an asthma exacerbation after a common cold (fever, sore throat, or rhinorrhea) in previous one week, indicating that the respiratory infection rather than the human respiratory colonization was usually observed prior to asthma exacerbation. The nasopharyngeal swab collection in these patients was performed within one week (average 4.4 days) after a common cold. The numbers of patients with viral infections, bacterial infections, both viral and bacterial infections, and no pathogen were 16 (33.3%), 9 (18.8%), 9 (18.8%), and 14 (29.2%), respectively (Fig 1). The dominant viruses were HRV A/B/C, RSV A, influenza A virus, and metapneumovirus (Table 2). Several simultaneous virus infections were detected in some patients: 3 viruses in 3 patients and 2 viruses in 3 patients. The method of bacterial detection was sputum culture (20.8%), multiplex PCR (41.7%) and both (37.5%), respectively. The major bacteria were *S*. *pneumoniae* and *H*. *influenzae* (Table 2). Several simultaneous bacterial infections were detected in a few patients: 3 bacteria in 1 patient, and 2 bacteria in 2 patients. Furthermore, co-infections of virus and bacteria were detected in 9 patients with no associations with specific viruses and specific bacteria (Table 2).

**Table 2:** Pathogens detected.

**Virus**	**n = 25**
Rhinovirus A/B/C	5
Influenza A	5
Respiratory syncytial virus A	5
Parainfluenza 3	4
Metapneumovirus	3
Enterovirus	3
Parainfluenza 2	3
Respiratory syncytial virus B	2
Parainfluenza 1	2
Coronavirus 22	1
**Bacteria**	**n = 18**
*S*. *pneumoniae*	9
*H*. *influenzae*	6
*CNS*	2
*M*. *catarrhalis*	1
*K*. *pneumoniae*	1
*H*. *parainfluenzae*	1
*B*. *pertussis*	1
*C*. *pneumoniae*	1
**Co-infection with viruses and bacteria (n = 9)**	
**Bacteria**	**Virus**
*K*. *pneumoniae*	Influenza A
*H*. *influenzae*	Influenza A
*S*. *pneumoniae*	Influenza A, RSV A
*S*. *pneumoniae*, *H*. *influenzae*	Metapneumovirus
*S*. *pneumoniae*	Metapneumovirus, Coronavirus 22
*H*. *influenzae*	Parainfluenza 2
*S*. *pneumoniae*, *H*. *influenzae*	Parainfluenza 3
*M*. *catarrhalis*	Parainfluenza 3, RSV A, B
*C*. *pneumoniae*	Parainfluenza 1

### Characteristics of pathogen-infected asthma exacerbation patients

Compared to pathogen-free patients, the infected patients were older and non-atopic and had later onset of disease, lower FeNO levels, higher IgE titers, and a higher incidence of comorbid sinusitis, COPD, and pneumonia (Table 3). There were no differences between the two groups in lymphocyte counts, CD4 absolute counts, and immunoglobulin levels (data not shown). After intensive treatment of the asthma exacerbation for one month, ACQ and FeNO levels decreased without any difference between the 2 groups. Compared to virus-free patients, the virus-detected patients were non-atopic type and high IgE titers (Table 3). Compared to bacteria-free patients, the bacteria-detected patients were high incidence of comorbid sinusitis and pneumonia (Table 3).

**Table 3:** Patients’ characteristics by pathogen detection (n = 48).

	Pathogen		Virus		Bacteria	
Characteristic	Positive	Negative	Positive	Negative	Positive	Negative
**n**	34	14	25	23	18	30
**Age (y)**	59.9	52.0	59.8	55.2	61.7	55.1
**Male (%)**	38.2	42.9	32.0	47.8	47.4	52.6
**Asthma onset age (y)**	41.0	32.0	40.4	36.2	43.0	35.6
**Duration of disease(y)**	19.9	21.0	20.3	20.0	19.7	20.5
**Atopic type(%)**	75.8	92.9	64.0* †	95.7	83.3	76.7
**Current smoker(%)**	38.2	35.7	28.0	47.8	44.4	33.3
**CD4 (/μL)**	338.2*	257.6	322.2	299.7	325.9	300.9
**CD8 (/μL)**	168.6	164.7	174.3	160.6	153.8	175.9
**Sinusitis (%)**	32.3	21.4	32.0	13.0	44.4* †	10.0
**Pneumonia (%)**	20.6	7.1	12.0	0.0	16.7*	0.0
**COPD (%)**	21.0	7.1	20.0	13.0	22.2	13.3
**IgE(IU/mL)**	649.8	340.5	722.9*	507.0	373.3	768.6
**ACQ**	3.6	3.8	3.6	3.7	3.6	3.7
**FeNO (ppb)**	42.6	69.4	34.2	68.1	52.2	50.5
**Post ACQ**	0.6	0.4	0.5	0.4	0.6	0.4
**Post FeNO (ppb)**	29.8	32.0	28.5	35.6	35.4	30.2

Post ACQ and FeNO levels were measured one month after asthma exacerbation.

*p < 0.05, Mann-Whitney U test

†p < 0.05, multiple logistic regression analysis.

### Pathogen comparison between stable outpatients and asthma exacerbation patients

There was no difference in the pathogen detection rate between inpatients and outpatients (Table 1). Viruses were detected in 50% of stable outpatients, and higher incidences of HRV A/B/C, RSV A, and metapneumovirus infections were observed in asthma exacerbation inpatients (Table 4). *H*. *influenzae* was observed even in stable asthmatic patients. Other bacteria other than *H*. *influenzae*, especially *S*. *pneumoniae*, were important in asthma exacerbation inpatients (Table 4).

**Table 4:** Pathogen detection between asthma inpatients (exacerbation) and stable outpatients (%).

	**Asthma attack inpatients**	**Stable outpatients**
**Virus**		
**Rhinovirus A/B/C**	10.4	5.0
**Influenza A**	10.4	10.0
**Respiratory syncytial virus A**	10.4	5.0
**Parainfluenza 3**	8.3	10.0
**Metapneumovirus**	6.3	0.0
**Enterovirus**	6.3	20.0
**Parainfluenza 2**	6.3	10.0
**Parainfluenza 1**	6.3	5.0
**Respiratory syncytial virus B**	4.2	5.0
**Coronavirus 22**	2.1	5.0
**Bacteria**		
***S*. *pneumoniae***	18.8* †	0.0
***H*. *influenzae***	12.5	30.0
***CNS***	4.2	0.0
***M*. *catarrhalis***	2.1	0.0
***K*. *pneumoniae***	2.1	0.0
***H*. *parainfluenzae***	2.1	0.0
***B*. *pertussis***	2.1	0.0
***C*. *pneumoniae***	2.1	0.0

*p < 0.05 by Pearson’s χ^2^ test

†p < 0.05, by multiple logistic regression analysis.

## Discussion

The recent asthma studies demonstrated the relationship between viral infections and the development of asthma [1, 2]. Even in the recent guidelines, there is few description of the association between bacterial infection and asthma [1–3]. In this study, infection with *S*. *pneumoniae* was the risk factor for adult asthma exacerbation in clinical practice.

The current guideline was developed using an evidence-based medicine analysis, which utilized several clinical studies. Patients with a heavy smoking history or comorbid COPD or pneumonia were usually excluded in almost all of the clinical asthma studies. However, many asthmatic inpatients have a history of heavy smoking and some have comorbid COPD or pneumonia in clinical practice. The results of the present study are useful for many practicing clinicians because the patients in the present study were not all non-smoking uncomplicated asthmatics.

It was found that viral infection, especially with IF, RSV, and HRV, was important for asthma exacerbation. These results are consistent with previous reports [5–7, 14]. Surprisingly, viruses were detected by multiplex PCR analysis in half of the stable asthmatic outpatients, indicating that several viruses were the common human respiratory microbes. Compared to stable asthmatic outpatients, higher incidences of HRV, RSV, and metapneumovirus infections were observed in asthma exacerbation inpatients, indicating that viral infection, especially with HRV, RSV, or metapneumovirus, plays an important role in adult asthma exacerbation in clinical practice. Prevention of infections with these viruses is important for decreasing asthma exacerbations in clinical practice. The relationship between viral infection and asthma exacerbation may be explained by TLR or ICAM-1 [20, 21, 23–27], but a higher incidence of smoking may affect viral infection in asthmatic patients in clinical practice.

Bacterial infection has not been the focus of previous studies of asthma exacerbation because patients who smoked or had COPD were usually completely excluded in usual clinical asthma studies. In the present study, there was a relationship between bacterial infection and asthma exacerbation in clinical practice. Considering the results of this practical, clinical study, comorbid sinusitis may be the most important factor related to susceptibility to bacterial infection in asthmatic patients. The presence of comorbid pneumonia may also affect the susceptibility of asthmatic patients to bacterial infection. Although *S*. *pneumoniae*, *H*. *influenzae*, and *M*. *catarrhalis* are the major bacterial infections in the respiratory tract, only *H*. *influenzae* was detected from stable asthmatic outpatients. These results indicate that bacteria other than *H*. *influenzae* contributed to asthma exacerbation in clinical practice. Early treatment with antibiotics, as well as steroid and bronchodilators, may be required for treatment of asthma exacerbation in these patients. It is important for clinicians to detect and treat bacterial infections in patients with asthma exacerbation in clinical practice. In the view point of the prevention of bacterial infection, pneumococcal vaccination is most important for asthmatic patients.

Co-infections with virus and bacteria were observed in 18.8% of asthma attack patients in this study. Wark et al. reported that the viral and bacterial co-infections increased the risk of readmission in asthmatic and COPD patients [14]. This is an important issue when taking care of these asthmatic patients.

The exacerbation group had a higher number of smokers versus the stable asthmatics in our study. The mechanism of this result may be explained by steroid resistance (via the histone deacetylase 2 pathway or overexpression of glucocorticoid receptor β) in severe smoking asthma. [29, 30]

There are some limitations in this study. Although 98% of asthma exacerbation inpatients had a history of a common cold before their asthma attack, a pathogen was detected from nasopharyngeal swabs in only 70% of patients. Overall, 15 major viruses and 6 major bacteria were evaluated by multiplex PCR analysis. It is possible that other minor pathogens may be involved in asthma exacerbations in clinical practice. Another possible reason for the low yield is inappropriate timing of the taking of nasal swabs. The pathogen causing the common cold may have disappeared at the time the nasal swab was taken in some patients. Another reason is that some patients may have only had a lower respiratory tract infection. The symptom from the patients with allergic rhinitis may be misinterpreted as being due to a respiratory infection. Furthermore, this study is not a result from longitudinal data. We checked the microbes only once on admission. If we check the microbes several times, we can clearly elucidate whether the detected microbe is infection or colonization. Several pathogens were different between stable outpatients and asthma exacerbation inpatients. However, only the detection rate of *S*. *pneumoniae* was statisitically significant because the number of stable outpatients was too small compared to asthma exacerbation inpatients. If we could get the larger number of outpatients, other pathogen may be related to asthma exacerbation.

In conclusion, several viral and bacterial infections were observed in patients with asthma attacks in clinical practice. Infection with *S*. *pneumoniae* was related to adult asthma exacerbation. In the future, a fast and easy method for the detection of pathogens is required for early treatment of viral or bacterial infections in asthma exacerbation inpatients.
